# Acute generalized exanthematous pustulosis induced by Moderna COVID-19 messenger RNA vaccine

**DOI:** 10.1016/j.jdcr.2021.08.013

**Published:** 2021-08-27

**Authors:** Anna Agaronov, Christopher Makdesi, Clifton Samuel Hall

**Affiliations:** a Touro University Nevada, College of Osteopathic Medicine, Henderson, Nevada; b Las Vegas Skin and Cancer Clinics, Las Vegas, Nevada

**Keywords:** acute, acute generalized exanthematous pustulosis, AGEP, COVID-19 vaccine adverse effects, COVID-19, cutaneous, Moderna COVID-19 mRNA vaccine, AGEP, acute generalized exanthematous pustulosis, mRNA, messenger RNA

## Introduction

COVID-19 caused by SARS-CoV-2 is responsible for the current pandemic. The US Food and Drug Administration recently granted emergency authorization of the Pfizer BioNTech and Moderna messenger RNA (mRNA) vaccines for use against COVID-19. Both vaccines utilize a novel technology, in which mRNA encoding the SARS-CoV-2 spike protein enveloped in lipid nanoparticles penetrates the cell membrane into the cytosol and produces a spike protein for subsequent antigen presentation and immune system activation.^1^ Although this vaccine technology is presumed to be safe through clinical trials, the full spectrum of adverse effects of mRNA vaccines has not yet been characterized. Nonetheless, a wide range of cutaneous adverse effects has been described with the use of Pfizer BioNTech and Moderna mRNA vaccines. Local immediate and delayed injection site reactions, urticaria, and morbilliform rashes are the most frequently reported adverse effects.^2^ We describe a rare cutaneous development of acute generalized exanthematous pustulosis (AGEP) in a patient following vaccination with the Moderna COVID-19 mRNA vaccine.

## Case report

**Fig 1:**
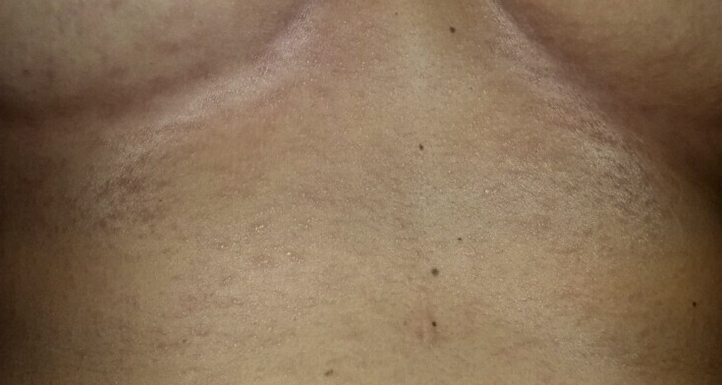
Numerous sterile pustules on an erythematous base resembling acute generalized exanthematous pustulosis within the inframammary folds and on the abdomen of the patient.

A 27-year-old Caucasian woman with a past medical history of anaphylactic reaction to tetracyclines presented for vaccination with the Moderna COVID-19 mRNA vaccine. Within a few hours of vaccination, subjective fever, fatigue, myalgias, and a mildly pruritic eruption under the breasts developed in the patient. Over the course of 3 days, the eruption spread to her abdomen, neck, and chest. The patient tried diphenhydramine and over-the-counter hydrocortisone 1% cream without relief. Physical examination showed variously sized erythematous and edematous papules and plaques on the abdomen, neck, and chest. Within the inframammary folds were numerous non–follicular-based pinpoint pustules (Fig 1). One week after the onset of the eruption, desquamation began in the inframammary region. The eruption completely resolved after 12 days. Due to the patient's uninsured status, the pustules were neither swabbed for culture nor biopsied for histopathologic examination. The diagnosis was made on a clinical basis. Given the extensive spread of the patient's eruption from the inframammary region to the chest, abdomen, and neck, a diagnosis of AGEP was favored over acute localized exanthematous pustulosis, a more localized variant of AGEP.

## Discussion

AGEP is a rare skin eruption characterized by an acute onset of numerous small, nonfollicular sterile pustules on an erythematous base. Clinically, AGEP is characterized by fever, with skin lesions typically beginning on the face or intertriginous zones (ie, axillae, groin, and inframammary folds) before disseminating over a few hours. Pruritus may also be present. Lesions normally last for 1 to 2 weeks, followed by superficial desquamation. More than 90% of cases of AGEP are due to the ingestion of drugs.^3^ Occasionally, AGEP may be induced by bacterial, parasitic, and viral infections.^4^, ^5^, ^6^ Recently, AGEP has been reported in patients with COVID-19 following treatment with hydroxychloroquine and cefepime.^7^^,^^8^ A late-onset AGEP-like vesicular cutaneous eruption has also been reported in a patient months after SARS-CoV-2 infection.^9^ Overall, only a handful of cases of AGEP after SARS-CoV-2 infection have been described in the literature.

Although AGEP is classically observed after drug ingestion, infection with SARS-CoV-2 may predispose certain patients to develop AGEP and AGEP-like pustular eruptions through its effect on the immune system. Notably, the inflammatory cytokine profile alterations and the cytokine storm in the setting of COVID-19 resemble the cytokine cascade changes in AGEP in many aspects.^10^ Herein, we report a case of AGEP in a patient following vaccination with the Moderna COVID-19 mRNA vaccine. An immune-mediated etiology for AGEP is plausible in this patient's vaccine-induced eruption, as the development of the eruption closely coincided with the administration of the vaccine. Additionally, the patient was in normal health with no recent symptoms and had no history of drug consumption in the 72 hours prior to vaccination. The presence of AGEP-like eruptions in patients with COVID-19 and in this patient following vaccination with an mRNA vaccine suggests a similar underlying etiology of immune activation leading to the development of AGEP.

Currently, AGEP is a seemingly rare cutaneous adverse effect following the Pfizer BioNTech and Moderna COVID-19 mRNA vaccines. As more of the population receives the vaccine, it is likely that cutaneous adverse effects will develop in more individuals, with AGEP and AGEP-like pustular eruptions potentially being reported more frequently. It is important for clinicians to be aware of this entity in their COVID-19–vaccinated patients, as this will allow a more prompt diagnosis and appropriate evaluation to exclude more serious cutaneous reactions. Further studies will be needed to discover the significance of this dermatologic finding in the setting of COVID-19 and COVID-19 vaccination.

## Conflicts of interest

None disclosed.
